# A Lifelong Smoker with Hypopituitarism: Rethinking the Hypothesis of a Tumor in the Hypophysis

**DOI:** 10.1155/2012/853568

**Published:** 2012-04-22

**Authors:** Nestoras Mathioudakis, Alfredo Quinones-Hinojosa, Roberto Salvatori, Shehzad Basaria

**Affiliations:** ^1^Division of Endocrinology & Metabolism, Johns Hopkins University School of Medicine, Suite 333, 1830 East Monument Street, Baltimore, MD 21287, USA; ^2^Department of Neurology and Neurosurgery, Johns Hopkins University School of Medicine, Cancer Research Building II, Room 253, 1550 Orleans Street, Baltimore, MD 21231, USA; ^3^Section of Endocrinology, Diabetes, and Nutrition, Boston Medical Center, Boston University School of Medicine, 2nd floor, 670 Albany Street, Boston, MA 02118, USA

## Abstract

Pituitary adenomas are the most common cause of a sellar mass. Metastases to the pituitary gland, a rare occurrence, may mimic benign pituitary adenomas. We report here a case of a 61-year-old woman with an 80-pack-year smoking history who presented with headache and diplopia. Visual field testing demonstrated bitemporal hemianopsia. Pituitary MRI revealed a 2.0 cm sellar mass impinging upon the optic chiasm. Hypopituitarism was present, with no evidence of diabetes insipidus. The patient was referred to our service for transsphenoidal resection of a presumed pituitary macroadenoma. As part of her preoperative evaluation, a chest radiograph was obtained, which showed a large hilar mass. In light of the patient's extensive smoking history, the differential diagnosis was expanded to include metastatic lesion to the sella. Transsphenoidal resection of the tumor was performed and histopathology revealed small cell carcinoma. The patient received chemotherapy, but died 18 months later due to widespread brain metastases. Although the presence of diabetes insipidus may help to discriminate between pituitary adenomas and metastatic lesions, this is not a sensitive finding. This case illustrates the need for maintaining a high index of suspicion for pituitary metastasis in patients with known risk factors for malignancy.

## 1. Introduction

The most common cause of an intrasellar lesion is a benign pituitary adenoma [1]. These tumors can be discovered incidentally or may become apparent from symptoms related to hormonal hypersecretion, hypopituitarism, diabetes insipidus, or mass effect on adjacent sellar structures. Rarely, a patient presenting with an apparent pituitary adenoma may, in fact, be harboring a malignancy that has metastasized to the sella. Metastatic lesions to the pituitary gland are uncommon and comprise less than 1% of pituitary tumors overall [2]. Distinguishing between a pituitary adenoma and a metastatic lesion on clinical grounds can be challenging given the overlap between symptoms of hypopituitarism and systemic malignancy. Furthermore, there are no clear-cut radiographic findings specific to metastatic lesions. One important clue to the diagnosis is the presence of diabetes insipidus, which is uncommon in pituitary adenomas but may occur in up to 70% of patients with metastatic disease [2]. As illustrated by this case, the sensitivity of this finding is clearly inferior to its specificity.

## 2. Case Presentation

**Figure 1:**
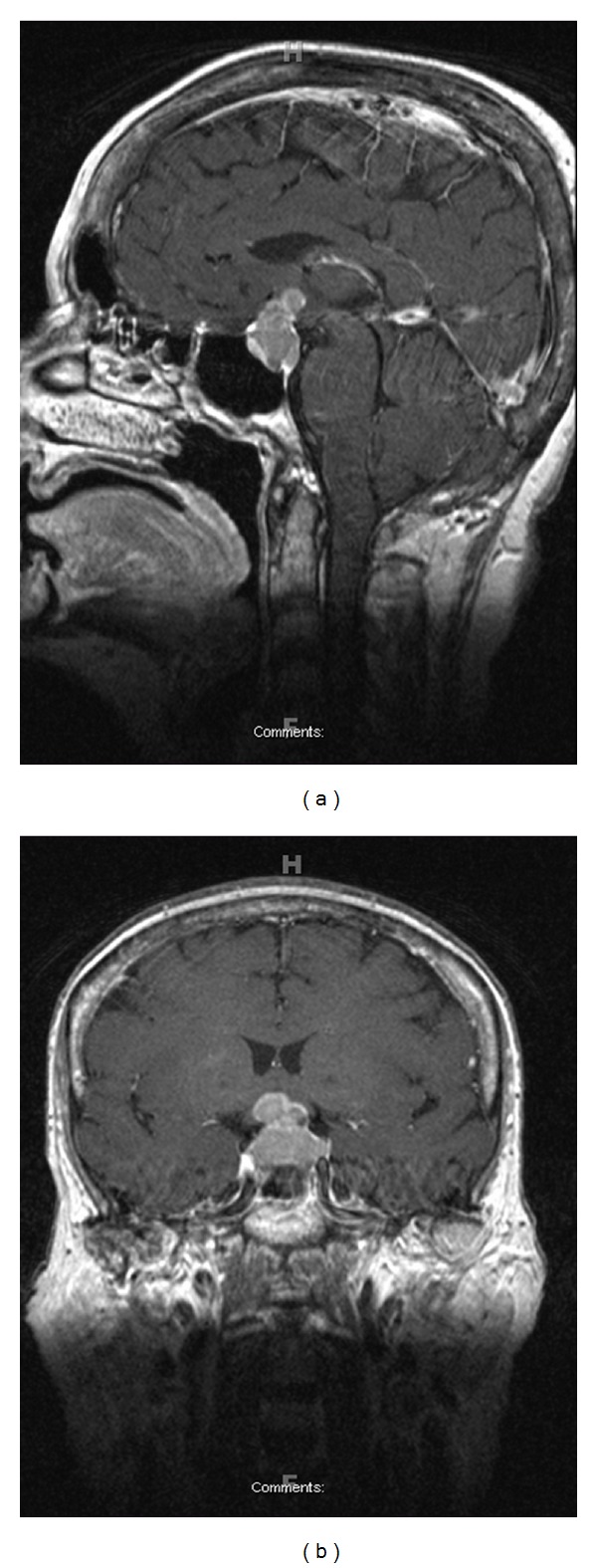
MRI brain, (a) coronal, (b) sagital: 2.0 × 1.2 × 1.1 cm bilobed homogeneous enhancing mass expanding the sella with compression of the optic chiasm.

We present the case of a 61-year-old woman with an eighty-pack-year smoking history who developed fatigue, anorexia, nausea, and a 11 kg weight loss over the course of one month. She complained of new-onset frontal headaches, requiring acetaminophen twice daily, and nausea, mitigated by prochlorperazine. Prior to these symptoms, she was very active and was employed at a steel mill. She initially attributed her symptoms to a viral illness, however within a few days she began to experience diplopia and diminished visual acuity and sought evaluation from her regular optometrist. She was noted to have raised intraocular pressure and was referred to our eye emergency room. An ophthalmology exam confirmed that she had raised intraocular pressure and bitemporal hemianopsia by perimetry. Magnetic resonance imaging demonstrated a 2.0 × 1.2 × 1.1 cm bilobed homogeneous enhancing mass expanding the sella with compression of the optic chiasm, suggestive of a pituitary adenoma (Figure 1).

The patient had a history of hypothyroidism subsequent to radioactive iodine therapy for Graves' disease. She was taking levothyroxine, and her previous thyroid function tests had been normal. She denied galactorrhea, bruising, acne, or striae. She had noted increasing thirst but denied polyuria. She felt that her shoes were slightly tighter, but she had no overt features of acromegaly. Trace pedal edema was noted. Although the patient had a history of hypertension, she had recently been experiencing postural hypotension off of all of her antihypertensive medications. Clinically, there was no evidence of a functioning pituitary tumor, though there was a clear concern about adrenal insufficiency.

A hormonal evaluation showed serum prolactin 92.1 ng/mL (normal 3–29), morning cortisol 4.6 mcg/dL (4.3–22.4), LH <0.2 mIU/mL, FSH 1.6 mIU/mL, estradiol <20 pmol/L (postmenopausal <115), IGF-I 133 ng/mL (114–492), TSH <0.01 mIU/mL (0.5–4.7), and free T4 1.36 ng/dL (0.8–1.7). Serum sodium was 135 mEq/L, and potassium was 4.6 mEq/L.

**Figure 2:**
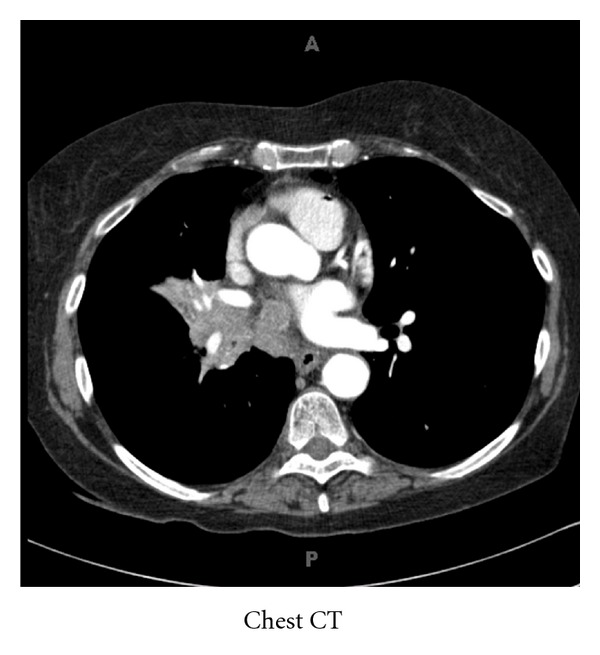
Chest CT: 6 cm right infrahilar mass with involvement of the subcarinal and paraesophageal nodes.

A preliminary diagnosis of a nonsecretory pituitary macroadenoma was made, with evidence of gonadotropin deficiency and probable central adrenal insufficiency. The hyperprolactinemia was attributed to stalk compression rather than a prolactinoma, as the prolactin level would be expected to be greater, typically above 200 ng/mL, in a prolactin-secreting tumor of this size. The possibility of the “hook effect” causing underestimation of the prolactin level was excluded by obtaining a diluted prolactin level [3]. Since the patient's vision was at risk, she was referred for surgery. During the course of her preoperative evaluation, a chest radiograph was taken which showed a 7 cm right hilar mass and nodules in the apex of the left lung. Given the patient's extensive smoking history, the differential diagnosis was expanded to include metastatic tumor to the sella. A follow-up computed tomography of the chest showed a 6 cm right infrahilar mass with involvement of the subcarinal and paraesophageal nodes as well as a 1.1 cm nodule in the left adrenal gland, highly suspicious for metastatic lung cancer (Figure 2). In the interim, she was started on glucocorticoid replacement for presumptive adrenal insufficiency because of worsening nausea and the development of abdominal pain. Her symptoms improved significantly after glucocorticoid replacement, and despite lack of confirmation with an ACTH stimulation test, her clinical response was strongly suggestive of central adrenal insufficiency. Two weeks later, she underwent transsphenoidal resection of the tumor for diagnosis and decompression of the optic chiasm. Histopathological examination of the resected pituitary lesion showed small cell carcinoma. The patient received radiation treatment to the sella and was started on chemotherapy. Initially, she had good response to single agent etoposide therapy but unfortunately presented 18 months later with widespread brain metastases and died shortly thereafter.

## 3. Discussion

 This case illustrates the importance of considering pituitary metastasis on the differential diagnosis of a sellar mass. Our patient was initially suspected of having a benign pituitary adenoma, by far the most common cause of a sellar lesion. Were it not for a routine chest radiograph obtained prior to transsphenoidal surgery, her diagnosis of lung cancer would have been made unexpectedly on pathology. Pituitary metastases are rare with an incidence of 0.14% to 28.1% of all brain metastases in autopsy series and 1% to 3.6% of patients with cancer [4, 5]. They account for less than 1% of pituitary tumors overall [6]. Breast and lung cancer are the most common primary sites; however, a wide range of tumors have been reported to metastasize to the pituitary [2]. Distinguishing between a benign pituitary adenoma and a metastatic lesion to the pituitary is a diagnostic challenge, both clinically and radiographically. These lesions may be asymptomatic; in fact, often the discovery of an underlying malignancy is made on pathology of the resected pituitary lesion. As with any sellar lesion, “mass effect” symptoms, including headache, visual field deficits, and ophthalmoplegia may result from expansion of the mass within the fixed space of the sella turcica, impingement upon the optic chiasm, or extension into the cavernous sinuses containing cranial nerves 3, 4, 5, and 6. The radiographic findings on CT or MRI are not specific for pituitary metastases and cannot reliably distinguish them from a benign adenoma [2]. Some MRI findings that favor metastasis include thickening of the pituitary stalk, loss of bright signal in the posterior pituitary, isointensity on T1 and T2 MRI images, invasion of cavernous sinus, and sclerotic changes surrounding the sella turcica [7, 8]. Benign pituitary tumors are generally indolent and slow growing; a rapidly growing tumor with extensive or aggressive infiltration into surrounding tissues should raise suspicion for pituitary metastasis [2]. Definitive diagnosis of pituitary metastasis requires transsphenoidal biopsy.

In the case of both primary and metastatic pituitary tumors, hypopituitarism may ensue from compression of the hormone-secreting cells of the anterior pituitary gland. Making the distinction between a pituitary adenoma and malignancy can be difficult, since the systemic complications of malignancy, including anorexia, weight loss, and vomiting, may overlap with anterior pituitary deficiencies, such as adrenal insufficiency. One helpful feature that points to the presence of a metastatic pituitary lesion is diabetes insipidus (DI), since only 1% of pituitary adenomas present with DI whereas up to 70% of metastases do so [2]. This difference has been attributed to the predilection for metastases to the posterior pituitary lobe due to its systemic blood supply and larger area of contact with the adjacent dura [5]. While the literature is replete with case reports of small cell lung cancer presenting with DI, the unusual feature of this case, and its inherent diagnostic challenge, is our patient's lack of symptoms referable to DI [7, 9–13]. Although untreated adrenal insufficiency can mask the hypernatremia of DI due to the development of an SIADH picture, treatment with glucocorticoids should expose the true source of the defect in water balance [14, 15]. An intact thirst mechanism and sufficient intake of free water may allow a patient with DI to compensate for the lack of ADH release and maintain normonatremia, so a normal serum sodium level, especially in the context of untreated adrenal insufficiency, does not exclude DI [5]. Our patient did not develop hypernatremia, polyuria, or polydypsia upon institution of glucocorticoid treatment, confirming the absence of DI. To further complicate matters, small-cell lung cancer may on occasion lead to the paraneoplastic syndrome of SIADH or even ectopic ACTH production, which could obscure defects at the level of the pituitary and hypothalamus in metastatic disease [16, 17].

Treatment options for metastatic pituitary lesions include surgical resection, radiation, and chemotherapy. The decision to perform surgery should be based on the clinical presentation, overall prognosis, and extent of the systemic disease. In our case, since a concurrent nonsecretory pituitary adenoma could not be ruled out conclusively on the basis of a lung biopsy alone, surgical decompression was performed with the intention of preserving vision and relieving her headaches, and also to establish a diagnosis. Indeed, after surgical debulking, her headaches and vision significantly improved. An alternative approach might have been transsphenoidal biopsy to confirm the metastasis from SCLC, followed by local radiation and systemic chemotherapy; however, biopsy of a sellar lesion is also high risk.

External radiation and/or chemotherapy may be used after surgery. The prognosis is generally poor, with some series showing a 10% 1-year survival [2]. Features related to poor outcome include age >65 at presentation, metastasis from small-cell lung cancer, and <1 year between the initial diagnosis of cancer and pituitary invasion [2].

The sudden onset of headache, ophthalmoplegia, or diabetes insipidus in a patient with risk factors for malignancy should raise suspicion for metastasis to the pituitary [2]. While diabetes insipidus, if present, may suggest the diagnosis, the absence of diabetes insipidus does not exclude metastatic disease. A high index of suspicion is thus required when evaluating a “pituitary tumor” in a patient with cancer risk factors.
